# An audit of patients with severe malnutrition secondary to eating disorders in Western Australia - a legacy of underinvestment in comprehensive care of youths and adults with eating disorders?

**DOI:** 10.1186/2050-2974-2-S1-O28

**Published:** 2014-11-24

**Authors:** Lisa Miller, Mary Bronson, Jan Fountaine, Charlotte Simmonds, Kiera James, Warren Ward, Adam Murphy, Sivanthe Senaratne, Robyn Lawrence

**Affiliations:** 1 Sir Charles Gairdner Hospital, Perth, Australia; 2 North Metro Area Mental Health Service, Perth, Australia; 3 Royal Brisbane & Women's Hospital, Brisbane, Australia

## 

From February 2012 until January 2014 an acute medical ward at a 600 bed tertiary hospital in Western Australia conducted an internal audit of youth and adult inpatient separations for patients admitted with complications of severe eating disorders. This cohort exhibited levels of severe malnutrition on par with or more severe than those managed in a specialist service in another state of comparable size, (the average BMI on admission was 12.4 range 9.8-15.8; n=12) and may reflect the fact that Western Australia is one of only two states / territories in Australia that does not have a publicly funded specialist adult inpatient eating disorders program.

Proposals for development of a state wide comprehensive specialist eating disorders service for youths and adults in Western Australia must take into account a potential legacy of decades of under investment in this area of health care, and potential barriers to optimising nutritional recovery within the confines of an activity based funding framework.

This abstract was presented in the **Service Initiatives** stream of the 2014 ANZAED Conference.

